# Effect of Community Education Program on Stroke Symptoms and Treatment on School and College Students from South India: A Longitudinal Observational Study

**DOI:** 10.3390/healthcare9121637

**Published:** 2021-11-26

**Authors:** Fayaz Khan, Riziq Allah Mustafa Gaowgzeh, Amer Al Saif, Mohamed Faisal Chevidikunnan, Ajith Soman, Aseel Mazi, Ejlal BinMulayh, Kirti Sundar Sahu, Haris Anjamparuthikal

**Affiliations:** 1 Department of Physical Therapy, Faculty of Medical Rehabilitation Sciences, King Abdulaziz University, Jeddah 21589, Saudi Arabia; rizikjo@yahoo.com (R.A.M.G.); aalsaif@kau.edu.sa (A.A.S.); mfaisal@kau.edu.sa (M.F.C.); aseel.f.mazi@gmail.com (A.M.); ejlal.bm.alansary@gmail.com (E.B.); 2 Department of Health Rehabilitation Science, College of Applied Medical Sciences, Shaqra University, Al Shaqra 11961, Saudi Arabia; ajithsoman78@gmail.com; 3 NITTE Institute of Physiotherapy, NITTE University, K S Hegde Medical Academy, Mangalore 575018, India; 4 School of Public Health and Health Systems, University of Waterloo, Waterloo, ON N2L 3G1, Canada; kirtisundar@gmail.com; 5 Department of Neurology, Government Medical College, Kottayam 686008, India; dr_aaharis@yahoo.com

**Keywords:** stroke, education, students, risk factors

## Abstract

Community awareness regarding stroke signs, risk factors, and actions that help reduce the risk and complications of stroke is poorly addressed, as it is thought to be the best approach to control and prevent stroke. Aim: To establish the awareness of stroke and its management among high school and college students using an educational intervention. A questionnaire was administered to students from five high schools and four colleges with different areas of focus, (arts, science and commerce), types (public, semi-public and private), and economic locations before and after an educational lecture on stroke. The lecture covered the following elements: stroke definition, signs, risk factors, actions, time window for thrombolytic therapy, and types of rehabilitation interventions. This study included 1036 participants, of whom 36.3% were male and 56.4% were high school students, and the mean age was 17.15 ± 1.29 (15–22) years. Before the lecture, 147 participants were unaware of a single sign of stroke, and 124 did not know the risk factors. After the intervention, 439 participants knew four signs of stroke, and 196 knew 12 risk factors. Female students had better knowledge about stroke signs (odds ratio (OR), 3.08; 95% confidence interval (95% CI), 2.15–4.43). Hypertension (52.7%) and weakness (59.85%) were the most known signs and risk factors. The proportion of students who selected traditional medicine as the mode of treatment decreased from 34.75% to 8.59% after the lecture. Other rehabilitation methods (e.g., physical therapy, occupational therapy, speech therapy and counseling) were chosen by more than 80% of the students. The results of the current study showed that the awareness on stroke risk factors and management among the school and college students can be significantly improved with regular educational interventions, and therefore stroke can be prevented to some extent.

## 1. Introduction

According to the World Health Organization (WHO), stroke and cerebrovascular disease were the major cause of death (15.2 million) in 2016 [1]. Death caused by cerebrovascular disease has increased to 88 per 100,000 per year according to the global burden of disease 2010 estimates [2]. The cumulative incidence and crude prevalence of stroke ranges from 105–152/100,000 people/year and 44.29–559/100,000 people, respectively [3]. The disease burden shows the significance of addressing this problem; moreover, in 2016 there were 116.4 million disability adjusted life years lost due to stroke worldwide [4].

India faces challenges in stroke management due to uncontrolled risk factors secondary to lack of awareness and medical facility access. The introduction of thrombolytic therapy within the first 4 h has reduced disability, but this effect is significantly affected by the distance to the hospital and, moreover, the lack of awareness of stroke signs [5,6,7].

Only 4.5% of people having a stroke call for help by themselves, while in 95.5% of stroke incidents, relatives or people around the patient call for help. These findings indicate that stroke awareness programs should include all community members [8]. Thus, community awareness regarding stroke signs, risk factors, and actions that help reduce the risk and complications of stroke [9], especially in India, should be encouraged, as it is thought to be the best approach to control and prevent stroke [5].

The majority of previous studies that focused on the detection of the baseline knowledge of stroke and its signs concluded that stroke awareness is poor among the general public [10,11,12,13]. Due to these shocking findings, our aim was to discern and improve the level of stroke awareness among high school and college students in India using an applicable intervention. Propagation of the awareness of stroke in the younger generation is ideal, as these individuals are the future of the country and may be able to take the necessary actions for their family and society. In addition, an evaluation of the awareness of the importance of rehabilitation after stroke, which has been poorly addressed, was included.

## 2. Materials and Methods

The current study adopted a longitudinal observational design and was approved by the Institutional Ethics Committee (Ref: NIPT/IEC/RP/008/2016) on 3 April 2017. The data were collected from five high schools and four colleges located in a mixture of rural and urban areas of south India. All participants were informed about the aim of the study, and formal written consent was obtained.

### 2.1. Participants

The participants included both male and female students from high schools (grades 11 and 12) and colleges (arts and science), except professional colleges. The institutions were randomly selected and categorized by type (public, semi-public, and private) and focus (science, art, and commerce). The participants were included in the study if they consented to participate in the study; the participants were required to be familiar with either the Malayalam or the English languages in which the awareness presentation was delivered. Students who were not willing to participate and who had to leave the school/college in between the session were excluded from the study.

### 2.2. Intervention

A 40-min awareness lecture was presented by a neuroscientist with a PhD in neuroscience and 15 years of experience in neurorehabilitation and neuroscience. The speaker used both English and a local language (Malayalam) according to the needs of the students, and he was proficient in both languages. The lectures were delivered from June 2017 to September 2017 at different institutions. They covered the following elements: stroke definition, signs, risk factors, actions, time window for thrombolytic therapy, and types of rehabilitation interventions.

### 2.3. Outcome Measure

A questionnaire which included three sections were used to measure outcomes. The English version of this questionnaire was validated in previous studies [14,15]. Subsequently, the questionnaire was translated to Malayalam to increase understanding for those sections where the participants were not proficient with the English language. Both versions were validated by five neuroscience experts. Content validity was performed with each item rated according to structure, clarity, and relevance and then selected per its level of agreement, for which 80% was set as the minimum percentage requirement. A pilot study was conducted with 30 participants to measure the amount of time required to complete and validate the questionnaire.

The participants completed the questionnaires immediately before and after the lecture in a single setting. The questionnaire was comprised of yes/no questions, multiple choice questions with one or more multiple answers, and fill in the blank questions. It consisted of three sections: section A contained demographic information, including age, gender, class, and family history of heart disease and stroke; section B contained stroke signs and risk factors; and section C contained the action to be taken when someone has a stroke, sources of information on stroke, the time window for thrombolysis, and the type of rehabilitation required by patients with stroke.

### 2.4. Statistical Analysis

Statistical analyses were performed with the R tool, and the Pearson chi square test was used for a univariate analysis. Descriptive data were presented as the percentage, mean, and standard deviation. For the bivariate analysis, participants who selected slurred speech and weakness, hypertension, diabetes, and smoking were considered knowledgeable of signs and risk factors of stroke, and were compared by gender, family history of heart disease, focus, and type of institution. Odds ratios and 95% confidential intervals (CIs) were calculated. A *p*-value < 0.05 was considered statistically significant.

## 3. Results

The study had a total of 1036 participants: 376 (36.3%) male students and 660 (63.7%) female students between the ages of 15 and 22 years (17.15 ± 1.29 years). Table 1 shows their demographic characteristics.

### 3.1. Family History

The sample included 17 (1.64%) participants who had a family history of both stroke and heart disease. A history of heart problems was noted in 123 (11.87%) participants, which is more than the number of participants with a history of stroke (60, 5.79%).

### 3.2. Signs of Stroke

Prior to the intervention, 147 (14.19%) of the participants did not know any stroke signs; 147 (14.19%) knew one sign; 17 (1.64%) knew five signs; and 6 (0.58%) knew six signs. After the intervention, 439 (42.37%) knew four signs; 293 (28.28%) knew five signs; and 69 (6.66%) knew six signs. Table 2 shows students’ knowledge about the signs of stroke.

### 3.3. Risk Factors

Before the lecture, 124 (11.97%) participants could not identify any of the risk factors; 91 (8.78%) identified one risk factor and one (0.1%) identified 12 risk factors; After the lecture, 196 (18.92%) participants identified 12 risk factors, and 157 (15.15%) identified 13 risk factors. Table 3 shows students’ knowledge about the risk factors of stroke.

### 3.4. Action

Calling a physician was the most frequently selected choice prior to the intervention (271 (26.16%)), followed by calling an ambulance (228 (22.01%)). After the intervention, 407 (39.29%) participants chose to call an ambulance.

### 3.5. Arrival Time to the Hospital

A total of 261 (25.19%) participants recommended 1 h as the optimum time to reach the hospital after the detection of stroke signs, and only eight (0.77%) suggested 4 h before the intervention. After the intervention, 555 (53.57%) participants recommended a timeframe within 4 h as the optimal time for a patient to arrive at the hospital after a stroke.

### 3.6. Source of Information

Knowledge about stroke was mainly gained from reading in both high school (216 (36.99%)) and college (161 (35.61%)) students, followed by Television/radio (210 (35.96%) for high school students and 153 (33.85%) for college students). After the lecture, health campaigns in 359 (61.47%) high schools and 333 (73.67%) college students followed by information from a physician in 278 (47.60%) high school students and 163 (36.06%) college students were the most common sources of information.

### 3.7. Rehabilitation Preference

Physical therapy (426 (72.95%)) and traditional treatments (253 (43.32%)) were the most frequently selected rehabilitation preferences in high school students and college students (248 (54.87%) and 107 (23.67%), respectively). After the lecture, the preferences for traditional treatment decreased to less than 10%, and other rehabilitation methods were selected by more than 75% of high school and college students.

Bivariate analysis: The results showed that female students were more knowledgeable about stroke signs than male students (odds ratio, 3.08 (2.15–4.43)), and students in semi-public schools were more knowledgeable about stroke signs (1.9 (1.1–3.23)) and risk factors (2.79 (1.88–4.13)) than other students. Additional odds ratios are listed in Table 4 and Table 5.

## 4. Discussion

A This study was conducted with 1036 high school and college students in south India to identify the level of knowledge about stroke and its management before and after an intervention. The current study results showed (i) a lack of stroke awareness among all students, which is comparable with other studies conducted in Australia [9], Greece [16], Germany [17], and the United Kingdom [18]; (ii) the ability of female students to identify stroke signs better than male students; and (iii) significant improvement in stroke awareness after an educational intervention.

### 4.1. Stroke Signs

In the current study, 14.19% of participants were unable to identify any of the stroke signs from a total of six signs. The characteristics of the sample in this study were better than those of the samples in other studies conducted in the same country, in which 22.51% of 942 were relatives of stroke patients [10], and 66% of 350 participants were unable to recognize any of the signs of stroke [11]. In the United Kingdom, 34% of 622 participants did not recognize any of the signs of stroke [18].

Weakness was the most noted sign in the current study (59.85%) and in previous studies that were conducted in northwest India (62.2%) [10], southern India (30%) [11], Nigeria (51.9%) [15], and Germany (40%) [17]. Our population had better knowledge of stroke signs than the populations of previous studies, and young age and being a student are associated with better recognition of the signs of stroke [9,15].

The results significantly improved after the education intervention. Female students in particular had better knowledge of stroke signs than male students, which is comparable with the findings of a study conducted in Saudi Arabia [19].

### 4.2. Risk Factors

In the current study, hypertension was the most frequently selected risk factor (52.7%), which supports the results of previous studies [10,11,15,17,18]. Being knowledgeable about hypertension as a factor is critical, as it is the causative factor of 54% of strokes in low and middle-income countries [20]. Knowledge about other risk factors, such as smoking, diabetes, family history of stroke, and obesity was lower in this study than in other studies [11,15]; this low level of knowledge could be improved by education, as was shown by the results of the current study.

### 4.3. The Source of Education

Reading was the preferable way to gain information for students. A study based in India showed that people gain knowledge from friends and relatives [10], which makes sense because socialization is a large part of Indian culture. Media was the source of education on stroke in Saudi Arabia [19]. Television was the common source in Nigerian students, teachers, and people above the age of 45 [15,21]. In Australia, the most common source was family members followed by television [9], whereas in Brazil, it was schools that made them aware of information on stroke [22]. Previous studies had populations with variable ages, which did not represent an appropriate method of education. Worthmann et al. [17] addressed this issue by categorizing the method of education by gender, age, and level of education. People with graduate school/college education responded better to advertisements on buses than other sources of information. Males, people under the age of 30, and people with university diplomas and doctorate degrees responded better to spots in the transportation system than other individuals. In general, flyers distributed in pharmacies were the best route of education in this population [17].

The preferred type of intervention depends on the characteristics of the targeted population, and financial support is another aspect that must be considered [17]. Campaigns that include mass media, posters, flyers and lectures, television advertisements, etc. tend to be expensive, and the learned knowledge or memory decays once the exposure to the information ends [17,23,24]. According to the current findings, educational lectures are less expensive and can significantly improve stroke awareness.

### 4.4. Arrival Time and Ambulance

Time is crucial in stroke management; this could be affected by ambulance services, an understating of the severity of stroke, the choice of heading to a community center rather than a stroke unit [11], and physicians’ knowledge of stroke [10]. In this study, only 0.8% of participants knew the optimal time window, and their first action was to call the physician; this was improved significantly after the intervention. Calling an ambulance should be addressed to decrease the arrival time, as 75% of participants used public transportation rather than the ambulance [11].

A previous study showed that individuals knew the actions they should perform when someone has a stroke when they were directly asked about it, but the responses were worse when individuals were asked about the actions after seeing only one of the symptoms without the mention of stroke [9]. Knowledge about stroke signs is not enough; taking an action when signs emerge is equally important.

### 4.5. Knowledge about Rehabilitation after Stroke

Many studies did not address knowledge about rehabilitation after stroke. The responses in the current study showed poor awareness of post-stroke rehabilitation; however, this was significantly improved after the intervention. Rehabilitation could be negatively affected by lack of knowledge, the accessibility of traditional medicine, and the fact that stroke units providing these services are private and located in urban areas, which are not accessible for many patients [25,26]. Moreover, a need for a multidisciplinary and comprehensive approach for the prevention and management of stroke should also be emphasized [27,28].

The study didn’t include the subjects from professional colleges like engineering, medical and allied health sciences, as the authors believed that the knowledge among these categories would be higher. However, a separate study is warranted for these categories. There should also be a long term follow-up to see whether the information delivered is retained, and strategies have to be implemented for the knowledge translation from these students.

## 5. Conclusions

A questionnaire pertaining to stroke awareness showed poor results among high school and college students living in India. However, their knowledge significantly improved after an educational intervention. Future research could additionally administer and evaluate follow-up questionnaires to reveal the extent of knowledge retained after a period of time and could compare the efficacy of an educational lecture with other modes of imparting stroke awareness and knowledge.

## Figures and Tables

**Table 1:** Demographic data.

Variables	Frequency	Percentage (%)
1 School	584	56.4
2 College	452	43.6
Institute type		
1 Government	200	19.3
2 Private	290	28.0
3 Semi government	546	52.7
Stream		
1 Science	519	50.1
2 Arts	199	19.2
3 Commerce	318	30.7
Sex		
1 Female	660	63.7
2 Male	376	36.3

**Table 2:** Knowledge about symptoms of stroke.

Signs	School (*n* = 584)		College (*n* = 452)		Total (*n* = 1036)	
	Pre (%)	Post (%)	Pre (%)	Post (%)	Pre (%)	Post (%)
Slurred Speech	280 (47.95)	530 (91.38) *	245 (54.21)	413 (91.38) *	525 (50.68)	943 (91.03) *
Weakness	359 (61.48)	510 (87.33) *	261 (57.75)	421 (93.15) *	620 (59.85)	931 (89.87) *
Headache	152 (26.03)	488 (83.57) *	95 (21.02)	413 (91.38) *	247 (23.85)	901 (86.97) *
Visual Problems	90 (15.41)	474 (81.77) *	53 (11.73)	373 (82.53) *	143 (13.81)	847 (81.76) *
Difficulty Understanding	168 (28.77)	223 (38.19) *	139 (30.76)	213 (47.13) *	307 (29.64)	436 (42.09) *
Shortness of Breath	190 (32.54)	60 (10.28)	157 (34.74)	55 (12.17)	347 (33.50)	115 (11.10)

* *p* < 0.05.

**Table 3:** Knowledge about risk factors of stroke.

Risk Factors	School (*n* = 584)		College (*n* = 452)		Total (*n* = 1036)	
	Pre (%)	Post (%)	Pre (%)	Post (%)	Pre (%)	Post (%)
Age	117 (20.04)	404 (69.18) *	78 (17.26)	248 (54.87) *	195 (18.83)	652 (62.94) *
Hypertension	317 (54.28)	479(82.02) *	229 (50.67)	373 (82.53) *	546 (52.71)	852 (82.24) *
Stress	252 (43.15)	325 (55.65) *	207 (45.80)	172 (38.06)	459 (44.31)	497 (47.98)
Smoking	171 (29.28)	531 (90.93) *	127 (12.26)	421 (93.15) *	298 (28.77)	952 (91.90) *
Cholesterol	259 (44.35)	502 (85.96) *	188 (41.60)	402 (88.94) *	447 (43.15)	904 (87.26) *
Diabetes	154 (26.37)	471 (80.65) *	114 (25.23)	348 (77.00) *	268 (25.87)	819 (79.06) *
Obesity	105 (17.96)	489 (83.74) *	101 (22.35)	385 (85.18) *	206 (19.89)	874 (84.37) *
Lack of Exercise	229 (39.22)	504 (86.31) *	159 (35.18)	399 (88.28) *	388 (37.46)	903 (87.17) *
Family History of Stroke	85 (14.56)	349 (59.76) *	49 (10.84)	249 (55.09) *	107 (10.33)	598 (57.73) *
Alcohol Use	183 (31.34)	534 (91.44) *	142 (31.42)	406 (89.83) *	325 (31.37)	940 (90.74) *
Poor Diet	217 (37.16)	476 (81.51) *	192 (42.48)	394 (87.17) *	409 (39.48)	870 (83.98) *
Increased Salt Intake	164 (28.09)	379 (64.90) *	86 (19.03)	333 (73.68) *	250 (24.14)	712 (68.73) *
Transient Ischemic Attack	99 (16.96)	370 (63.36) *	49 (10.84)	261 (57.75) *	148 (14.29)	631 (60.91) *

* *p* < 0.05.

**Table 4:** Knowledge about symptoms of stroke in different variables.

Variables		Pre	Post
Sex	Male	107	290
	Female	288	602
	OR (CI %)	1.95 (1.49–2.56)	3.08 (2.15–4.43)
Family history of heart disease	Yes	56	109
	No	339	783
	OR (CI %)	1.41 (0.97–2.07)	1.29 (0.74–2.42)
Stream (Science vs. Arts)	Science	223	460
	Arts	71	167
	OR (CI %)	0.73(0.52–1.02)	0.66 (0.42–1.07)
Stream (Science vs. Commerce)	Science	223	460
	Commerce	101	265
	OR (CI %)	0.8 (0.7–0.9)	0.8 (0.65–0.97)
School vs. College	School	229	493
	College	166	399
	OR (CI %)	1.11 (0.86–1.43)	0.71 (0.5–1.03)
Public vs. Private	Public	72	170
	Private	123	242
	OR (CI %)	1.09 (0.76–1.58)	1.62 (0.9–2.91)
Public vs. Semi Public	Public	72	170
	Semi Public	200	480
	OR (CI %)	1.15 (0.82–1.6)	1.9 (1.1–3.23)

**Table 5:** Knowledge about risk factors of stroke in different variables.

Variables		Pre	Post
Sex	Male	33	222
	Female	39	499
	OR (CI %)	1.53 (0.94–2.48)	0.46 (0.35–0.61)
Family history of heart disease	Yes	6	92
	No	66	629
	OR (CI %)	0.66 (0.06–0.1)	1.34 (0.88–2.09)
Stream (Science vs. Arts)	Science	33	410
	Arts	14	114
	OR (CI %)	1.11 (0.56–2.0)	0.35 (0.25–0.50)
Stream (Science vs. Commerce)	Science	33	410
	Commerce	25	197
	OR (CI %)	1.12 (0.85–1.46)	0.65 (0.6–0.75)
School vs. College	School	39	416
	College	33	305
	OR (CI %)	0.91 (0.56–1.46)	1.19 (0.91–1.55)
Public vs. Private	Public	15	137
	Private	19	202
	OR (CI %)	1.59 (0.78–3.22)	1.13 (0.72–1.78)
Public vs. Semi Public	Public	15	137
	Semi Public	38	382
	OR (CI %)	1.2 (0.66–2.18)	2.79 (1.88–4.13)

## Data Availability

Data available on request.
